# Performance on a pattern separation task by Alzheimer’s patients shows possible links between disrupted dentate gyrus activity and apolipoprotein E ∈4 status and cerebrospinal fluid amyloid-β42 levels

**DOI:** 10.1186/alzrt250

**Published:** 2014-04-15

**Authors:** Keith A Wesnes, Peter Annas, Hans Basun, Chris Edgar, Kaj Blennow

**Affiliations:** 1 Wesnes Cognition Ltd., Little Paddock, Streatley Hill, Streatley on Thames RG8 9RD, UK; 2 Department of Psychology, Northumbria University, Newcastle, UK; 3 Centre for Human Psychopharmacology, Swinburne University, Melbourne, VIC, Australia; 4 Bracket, Wayne, PA, USA; 5 BioArctic Neuroscience AB, Stockholm, Sweden; 6 Formerly, Bracket, Goring on Thames RG8 9RD, UK; 7 Clinical Neurochemistry Laboratory, Sahlgrenska University Hospital, Mölndal, Sweden

## Abstract

**Introduction:**

Emerging evidence suggests that decreased adult hippocampal neurogenesis represents an early critical event in the course of Alzheimer’s disease (AD). In mice, adult neurogenesis is reduced by knock-in alleles for human apolipoprotein E (ApoE) ∈4. Decreased dentate gyrus (DG) neural progenitor cells proliferation has been observed in the triple-transgenic mouse model of AD (3xTg-AD); this reduction being directly associated with the presence of amyloid-β (Aβ) plaques and an increase in the number of Aβ-containing neurons in the hippocampus. Cognitive tasks involving difficult pattern separations have been shown to reflect DG activity and thus potentially neurogenesis in both animals and man. This study involved the administration of a pattern separation paradigm to Alzheimer’s patients to investigate relationships between task performance and both ApoE status and cerebrospinal fluid (CSF) Aβ42 levels.

**Methods:**

The CDR System pattern separation task involves the presentation of pictures that must later be discriminated from closely similar pictures. This paper presents pattern separation data from 66 mild to moderate AD patients, of which 50 were genotyped and 65 in whom CSF Aβ42 was measured.

**Results:**

ApoE ∈4 homozygotes were not compromised on the easy pattern separations compared with the other patients, but they were statistically significantly poorer at the difficult separations. In all patients CSF Aβ42 correlated significantly with the ability to make the difficult discriminations, but not easier discriminations. Pattern separation speed correlated negatively with CSF Aβ42, and thus the association was not due to increased impulsivity.

**Conclusions:**

These are, to our knowledge, the first human pattern separation data to suggest a possible genetic link to poor hippocampal neurogenesis in AD, as well as a relationship to Aβ42. Therapies which target neurogenesis may thus be useful in preventing the early stages of AD, notably in ApoE ∈4 homocygotes.

## Introduction

The human hippocampus supports the formation of episodic memory without confusing new memories with old ones [1]. To accomplish this, the brain must disambiguate memories - and this is now widely recognized as a key role of the dentate gyrus (DG). Convergent lines of evidence from neuroanatomical, electrophysiological, behavioral and human brain imaging studies suggest a crucial role for the DG in the formation of new episodic memories, by transforming similar experiences or events into discrete non-overlapping representations, a process known as “pattern separation” [2]. Schmidt *et al*. [1] write: “Over the course of the last 20 years, the overwhelming majority of data have supported the notion of the DG as a critical mediator of pattern separation within the hippocampal formation” (page 57).

Human evidence from functional Magnetic Resonance Imaging (fMRI) work has accumulated over the last few years linking the DG and CA3 subregions to performance on a pattern separation paradigm. Kirwan and Stark [3] used a continuous recognition paradigm, conducted during fMRI scanning, in which a series of pictures of everyday objects was presented and the participants had to classify each picture as new, similar or previously presented. Some of the pictures were repeated across trials and some pictures were presented that were very similar but not identical to previously presented objects. These latter objects, termed lures, were hypothesized to result in increased interference and an increased need for pattern separation due to the overlapping object features. fMRI activity in the hippocampus was found to distinguish between correctly identified old stimuli, correctly rejected similar lure stimuli, and false positive responses to similar lures. A further study from this group investigated fMRI activity in eight medial temporal lobe subregions when participants viewed these same stimuli; that activity, consistent with a strong bias towards pattern separation, was observed in and limited to the DG/CA3, which the authors interpreted as compelling evidence of a key role of this region in pattern separation [4]. As DG activity is known to decline with human aging, Toner *et al*. [5] used the continuous recognition paradigm of Kirwan and Stark to evaluate the effects of age on pattern separation. They contrasted the pattern separation performance of 20 young adults with 20 adults aged over 65 years, finding that the older adults were more likely to commit false positive errors and identify lure stimuli as old. However, there were no significant age-related differences in correctly identifying first stimuli as new or repeated stimuli as old. This was interpreted to indicate that in non-demented adults, age-related changes in hippocampal subregions may result in less efficient pattern separation. Wesnes [6] evaluated data from 3,067 healthy individuals aged from 18 to 87 years on another pattern separation paradigm - the Clinical Dementia Rating (CDR) System picture recognition task [7]. In this task, a series of pictures of everyday objects and scenes is presented to the participant, followed around 10 to 12 minutes later by a series containing these pictures mixed with very similar ones. The data confirmed Toner *et al*.’s finding that older participants were selectively poorer at correctly rejecting the closely similar pictures (lures), performing at the level of younger participants when correctly identifying previously presented pictures. This finding was extended by the demonstration that from the 20s onwards, the ability to make the difficult discriminations (correctly identify the lures as new) declined decade by decade from the 20s onwards. Again no such deterioration was seen for the ability to correctly identify the previously presented pictures (pattern completion). One difference between the paradigms is that the pattern separation task used by Toner and previous groups involves continuous recognition, whereas the CDR System task has a gap of 10 to 12 minutes before the recognition phase begins. The similarity of the findings in the two studies suggests that the two paradigms are assessing a common cognitive process. Yassa *et al*. [8] studied young and elderly adults in the continuous recognition pattern separation paradigm, and replicated Toner *et al*.’s finding that the elderly were poorer at correctly rejecting previously presented pictures. They also employed a Mnemonic Similarity Task in which 128 pictures of common objects were presented at the rate of one every 2.5 seconds. After a delay of several minutes, they were then shown 64 of the previous objects, 64 new objects and 64 objects that were similar but not identical to the original objects (lures). As in the previous study, the older participants were less likely to correctly identify the similar objects, and further analysis indicated that the degree of difference of the similar objects had to be greater in order for the performance of the elderly to match that of the young. The findings indicate that continuous recognition paradigms identify more similar age-related changes than paradigms in which recognition is delayed. The duration of the task employed by Yassa *et al*. was around 18 minutes, which is slightly longer than the CDR System paradigm. Overall, the comparable patterns of results between the three paradigms suggest that the CDR System task is measuring a behavioral phenomenon similar to continuous recognition tasks.

Yassa *et al*. [9], using the continuous pattern discrimination task, found that patients with amnestic Mild Cognitive Impairment (aMCI) performed at the same level as normal age-matched controls in identifying previously presented items, but were poorer in correctly identifying lure items [8]. A similar finding was identified with aMCI patients using the CDR System pattern separation task [6], again suggesting that the two paradigms assess similar behavioral phenomena. Stark *et al*. [10] identified age-related declines in difficult pattern separations in healthy adults, replicating earlier findings [5,6]. However, when the older adults (60 years and over) were separated based on their performance on delayed word recall in the Rey Auditory Verbal Learning Test, those performing more poorly also showed a deficit in difficult pattern separations, to a level comparable to patients with aMCI. However, in this study, the aMCI patients were poorer than age-matched controls on both easier and difficult pattern separations. Ally *et al*. [11] studied aMCI patients and mild Alzheimer’s disease (AD) patients in the pattern separation task used previously (for example, [3]). They identified that pattern separation was impaired in aMCI and further impaired in mild AD, whereas pattern completion was unaffected in aMCI but impaired in mild AD.

The seminal discovery, that in humans the DG retains its ability to generate neurons throughout life [12], has raised the possibility that therapies could be developed to protect or promote such neurogenesis, as it deteriorates due to ageing, insult and disease. Ageing itself results in a marked decline in adult brain neural stem/progenitor cells and neurogenesis, with concomitant impairments to cognitive functions [13]. Another major milestone occurred on 28 August 2013 when a team of Columbia University Medical Center researchers, led by Nobel laureate Eric Kandel, published a paper in the online edition of *Science Translational Medicine,* which identified the deficiency of a protein (RbAp48) in the hippocampal DG to be a significant contributor to age-related memory loss, and also demonstrated that this memory loss is reversible [14]. This finding builds on previous proposals that memory disturbances both in normal ageing and the early stages of Alzheimer’s disease could result from degenerative changes which compromise neurogenesis in the DG [15].

Lazarov and Marr [16] write: “While a massive and progressive neuronal loss in specific areas such as the hippocampus and cortex unequivocally underlies cognitive deterioration and memory loss in Alzheimer's disease, noteworthy alterations take place in the neurogenic microenvironments, namely, the subgranule layer of the dentate gyrus and the subventricular zone. Compromised neurogenesis presumably takes place earlier than onset of hallmark lesions or neuronal loss, and may play a role in the initiation and progression of neuropathology in Alzheimer's disease” (page 267). In mice, adult neurogenesis can be reduced by knock-out alleles for apolipoprotein E (ApoE), as well as by knock-in alleles for human ApoE ∈4 [17]. Decreased DG neural progenitor cell proliferation has been observed in 3xTg-AD mice; this reduction being directly associated with the presence of Aβ plaques and an increase in the number of Aβ-containing neurons in the hippocampus [18].

Overall, behavioral evidence is accumulating from pattern separation paradigms that DG activity and, thus, possibly neurogenesis, declines in human normal ageing and does so at a greater rate in aMCI and mild AD. The purpose of the present analysis was: (1) to use data from the CDR System pattern separation task to compare the performance of AD patients on the task to that of age-matched controls; (2) to determine whether in AD, a profile could be detected which was consistent with preclinical findings of a linkage of ApoE ∈4 to compromised DG neurogenesis [17]; and (3) to determine whether AD patients’ performances on the task could provide evidence consistent with the established relationship of Aβ to decreased DG neural progenitor cells [18].

## Methods

### Participants

Sixty six (31 female) mild to moderate Alzheimer’s disease patients (Diagnostic and Statistical Manual of Mental Disorders, Fourth Edition (DSM-IV)), aged from 59 to 100 years (mean 76.6, SD 7.5), with mean *mini–mental state examination* (MMSE) scores of 23.9 (SD 3.6; range 16 to 30) and Alzheimer’s Disease Assessment Scale – cognitive subscale (ADAS-cog) scores of 14.2 (SD 6.8; range 1.33 to 31.7) participated in this study. Participants were on stable, continuous treatment with acetylcholinesterase inhibitors, but were not included if treated with memantine, lithium, warfarin or recently started (less than three months) central nervous system active substances (for example, anti-depressants and neuroleptics). Participants with other concomitant diseases which might interfere with the study objectives were also excluded. All participants provided signed, written informed consent as well as that of a relative⁄legal representative, and were deemed able to comply with the study procedures. The study was conducted according to the provisions of the Helsinki Declaration and was approved by the Ethics Committees of the Universities of Lund and Uppsala and at the Karolinska Institute, Stockholm, Sweden.

Control data were taken from the CDR System database and comprised 238 individuals (104 female) aged from 71 to 91 (mean age 76.9, SD 4.1), with mean MMSE scores of 28.9 (SD 1.1).

### Procedure

The study was a three-center (Malmo University Hospital, Huddinge University Hospital and Uppsala University Hospital), open-study conducted over 18 months between 2004 and 2005. Participants completed a maximum of three hospital visits. At an initial visit, written informed consent was obtained, and at a subsequent visit within four weeks, participants completed various tests, including the CDR System pattern separation and underwent cerebrospinal fluid (CSF) and blood sampling.

#### CSF and blood sampling

CSF (8 ml) was collected according to hospital routine and transferred to polypropylene test tubes. Cellular material was pelleted by centrifuge within 30 minutes (1,500 × g, + 4°C for 15 minutes), then frozen in 2 ml aliquots and stored at -70°C [19]. Genotyping was conducted from the blood samples.

#### Cognitive testing

The CDR System [6,7,20,21] Picture Recognition Task was used. In this task a series of 14 pictures of everyday scenes and objects was presented on the screen at the rate of one every four seconds for the participant to remember. Around 12 minutes later the 14 pictures were re-presented mixed with 14 novel pictures which were individually matched to the original pictures to be closely similar. For each picture the participant was required to decide whether it was either an originally presented picture or a novel picture, by pressing a YES or NO button as appropriate, and as quickly as possible. The percentage of correctly identified previously presented pictures (pattern completion) was recorded as well as the percentage of correctly rejected novel but closely similar pictures (pattern separation). For each type of discrimination, speed of response in milliseconds was also recorded.

The participants also performed three CDR System tests of attention: simple reaction time, choice reaction time and digit vigilance [20,21]. The speed scores from these three tasks are combined to provide an index of focussed attention and an information process named Power of Attention, which has been validated by factor analysis [21].

### Statistics

All analyses were performed using the SAS® System (SAS Institute, Cary, North Carolina, USA). Differences between AD participants and controls were analyzed using mixed model analyses of covariance (ANCOVA). For each ANCOVA, a fixed term was fitted to the model for type (control or participant) and a random term for participants. Age was fitted as a covariate. Differences on various measures between the ApoE genotypes were evaluated by mixed model ANCOVAs. For each ANCOVA, a fixed term was fitted to the model for the ApoE genotype and a random term for participants. Age was fitted as a covariate. Other exploratory covariates were fitted and reported. Associations between measures were evaluated by calculating Spearman's Rho correlation coefficients.

## Results

Although some findings from this study have been reported previously [20], none of the data presented here has previously been published. CDR System pattern separation data were available for all participants, CSF Aβ42 levels for 65 participants and ApoE genotyping for 50.

### Comparisons to controls

There were highly significant differences on the task for the ability to correctly identify the original pictures (F(1,301) = 124.4, *P* <0.0001) and the ability to identify the closely similar pictures (F(1,301) = 68.4, *P* <0.0001). For the original pictures, the deficit in the AD patients compared to controls was 16.1% (95% confidence intervals 13.3 to 19), while for the closely similar pictures the deficit was 15.8% (95% confidence intervals 12 to 19.5).

### ApoE genotype

**Figure 1:**
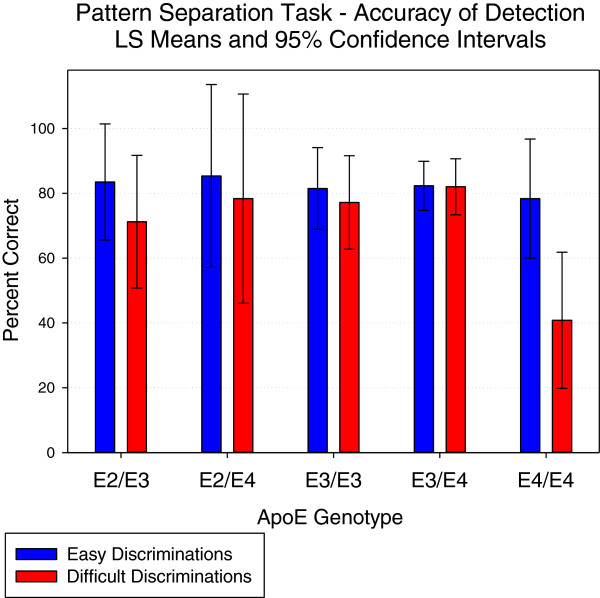
**Comparison of the scores of the ApoE genotypes on the Pattern Separation Task.** ApoE, apolipoprotein E; CDR, Clinical Dementia Rating; DG, dentate gyrus.

There were five ApoE genotypes (see Table 1). Inspection of the accuracy scores indicates that the ApoE ∈4/∈4 genotype scored notably lower than the other genotypes on the difficult pattern separations but not on the easier separations. The difference on the difficult pattern separations between the ∈4/∈4 genotype and the other four genotypes was 33.4% on average. However, the ∈4/∈4 genotype was younger than the other four genotypes by five to seven years, and age therefore was fitted to the ANCOVA models as a covariate. The analysis found a significant main effect of genotype for the difficult pattern discriminations (F(4.44) = 3.5, *P* = 0.0168) but not the simple ones (F(4.44) = 0.1, *P* = 0.99). The least squares means from the ANCOVAs of the easy and difficult separations are plotted in Figure 1 with 95% confidence intervals (CI). Focusing on the difficult pattern separations, it can be seen that the CI for the ∈4/∈4 genotype do not overlap the least squares means of the other four genotypes, nor do the CIs of the other four genotypes overlap the least squares mean of the ∈4 homozygotes. The Cohen’s d effect sizes of the differences between the ∈4/∈4 genotype and the other groups ranged from 1.3 and 1.8, and thus exceeded the convention of large effects for this measure (0.8), making the differences of clear clinical relevance.

**Table 1:** Demographics, cognitive test scores and CSF biomarker levels for the five genotypes (means with SD)

**ApoE** **Genotype**	**n**	**Age** **Years**	**MMSE**	**ADAS-Cog**	**Picture recognition-pattern separation task**				**Power of attention**	**Aβ42**	**P-tau**	**T-tau**
					**Difficult separations**		**Easy separations**		**msec**			
					**% Correct**	**Speed**	**% Correct**	**Speed**				
∈**2/**∈**3**	5	77 (10)	22 (5)	15 (8)	70 (28)	2,981 (3,137)	81 (15)	1,384 (443)	1,555 (296)	715 (113)	63 (17)	457 (147)
∈**3/**∈**3**	10	75 (9)	23 (4)	15 (10)	77 (26)	2,393 (1,450)	81 (17)	1,774 (1639)	1,621 (596)	536 (316)	76 (27)	555 (214)
∈**4/**∈**2**	2	75 (10)	26 (1)	9 (1)	79 (0)	1,078 (280)	86 (10)	1,099 (262)	1,430 (326)	439 (256)	66 (35)	535 (274)
∈**4/**∈**3**	28	76 (6)	25 (4)	13 (7)	82 (21)	1,830 (1,197)	82 (24)	1,483 (972)	1,504 (459)	414 (179)	99 (45)	748 (395)
∈**4/**∈**4**	5	70 (6)	22 (3)	19 (3)	44 (24)	4,263 (5,567)	84 (11)	2,132 (1,621)	1,579 (457)	295 (60)	97 (12)	769 (146)

ADAS-cog, Alzheimer’s Disease Assessment Scale – cognitive subscale; ApoE, apolipoprotein E; CSF, cerebrospinal fluid; MMSE, mini–mental state examination.

To determine whether this effect could simply be due to overall differences in cognitive ability between the five cohorts, the two standard scales were also subject to ANCOVA, but no significant main effects of genotype were identified for either MMSE (*P* >0.3) or ADAS-cog (*P* >0.49). To examine the possibility that the observed differences in difficult pattern separations may be related to poorer visual information processing in the homozygotes - the measure of focused attention and information processing (Power of Attention) was also fitted as a covariate, but the main effect of genotype still remained (*P* = 0.0181). Finally, there were clear differences between the genotypes in the levels of Aβ42, which were highly significant (*P* = 0.0034), although when Aβ42 was fitted as a covariate, the main effect remained (*P* = 0.0157).

A final consideration was that the response style may have been different in the ∈4/∈4 genotype; these participants, for example, may have been more impulsive, and made less correct rejections of the different but similar pictures by responding more quickly. ANCOVAs run on the speed scores found no overall effects for either the easy discriminations (F(4,43) = 0.8, *P* = 0.5326) or the difficult ones (F(4,44) = 1.81, *P* = 0.1433). However, paired-t comparisons did identify a slower speed of the ∈4/∈4 group in making the difficult discriminations compared to the ∈4/∈3 group (*P* = 0.0168), with trends for the ∈3/∈3 (*P* = 0.0877) and ∈4/∈2 groups (*P* = 0.0706). As can be seen in Table 1, the response times in the ∈4/∈4 group were between 43% and 295% longer than the other genotypes. This indicates that not only did the ∈4/∈4 group find the difficult discriminations harder, and thus made less correct rejections, but that they also took longer to do so as a consequence, though the increased reaction times are only of marginal statistical reliability. What is clear is that for the ∈4/∈4 group, accuracy on the difficult discriminations was not being compromised at the expense of speed of responding.

### Relationship of CSF Aβ42 levels to the performance on the Pattern Separation task

Although Aβ42 levels were not responsible for the differences between the five genotypes on difficult pattern separations, transgenic mice 3xTg-AD (who have greater Aβ pathology) show reduced DG neural progenitor cell proliferation, this reduction being directly associated both with the presence of Aβ plaques, and also an increase in the number of Aβ-containing neurons in the hippocampus [18]. To determine whether, for the 65 participants whose Aβ42 levels had been measured, there was a relationship between Aβ42 and the performance on the pattern separation task, correlations were performed. These are summarized in Table 2, from which it can be seen that higher levels of Aβ42 were significantly associated with greater accuracy in the 65 participants on the difficult separations, and also with faster response to these stimuli. In contrast, there was no significant association with the accuracy of the easy discriminations. Although the coefficients are modest, they do exceed the convention for Pearson coefficients of medium effect sizes (0.3) and are also of clinical relevance.

**Table 2:** Pearson Correlations of CSF biomarkers with performance measures from the Pattern Separation Task

**Task measures**	**Aβ42**		**T-tau**		**P-tau**	
	**Rho**	***P***	**Rho**	***P***	**Rho**	***P***
**Easy separations % correct**	0.13	0.312	0.03	0.81	0.09	0.47
**Difficult separations % correct**	0.32	0.0106	-0.09	0.46	-0.05	0.71
**Easy separations speed**	-0.3	0.0166	0.19	0.14	0.09	0.46
**Difficult separations speed**	-0.38	0.0021	0.05	0.67	-0.05	0.7

CSF, cerebrospinal fluid.

Although no expectation was made of relationships between Tau, the correlations are presented for illustrative purposes in Table 2, but as can be seen, there was little association.

## Discussion

In the present study, Alzheimer’s patients showed significant deficits on the aspects of the CDR System Picture Recognition task which reflected pattern completion (correctly identifying original pictures), as well as the part which reflected pattern separation (correctly identifying the closely similar pictures). The deficits on the two aspects of performance were very close in magnitude. This finding is consistent with that of Ally *et al*. [11]. However, the AD patients who were ApoE ∈4 homozygotes were statistically significantly poorer at the pattern separation part of the task, but not on the ability to recognize the previously presented pictures, which reflects pattern completion. These participants also showed numerically slower speed in rejecting the closely similar pictures. This was thus an unequivocal impairment: poorer accuracy being accompanied by slower speed. The effect sizes of these impairments were notable and of clear clinical and everyday relevance. When correlations were performed between these task measures and CSF Aβ42, a significant association was detected between the ability to correctly reject the closely similar pictures and levels of Aβ42, which exceeded a medium effect size, and thus also could be considered clinically relevant. In animals, performance on pattern separation tasks is related to DG activity and neurogenesis, with reduced ability to make difficult discriminations (pattern separation) being associated with disruption to the DG and impaired neurogenesis [2,22]. Further, animals with strongly reduced levels of dentate gyrus neurogenesis have been shown to be impaired in a hippocampus-dependent object recognition task [23]. These data therefore suggest that the ApoE ∈4 homozygotes have greater impairments to the DG than the other ApoE groupings. Although this relationship did not disappear when Aβ42 levels were used as covariates, separate analyses identified Aβ42 levels to be related to the ability to make difficult separations. This suggests two possibly independent risk factors of reduced DG activity in AD: having two ApoE ∈4 alleles or having lowered CSF levels of Aβ42.

Overall, to our knowledge, these are the first pattern separation data from Alzheimer’s patients which provide evidence that pattern separation deficits are related both to ApoE ∈4 genotypes and CSF Aβ42. The data are consistent with previous findings of compromised performance on this measure in normal aging and, also, the greater impairment seen in the Alzheimer’s disease prodrome - amnestic MCI. While until recently the relationship to pattern separation tasks, DG activity and neurogenesis has not been confirmed in humans, the data just published by Mandel and colleagues identified the RbAp48 protein to be specifically lower in human *post mortem* dentate gyrus tissue [14]. The group then went on to manipulate the protein in mice and found clear relationships to age-related memory decline (also associated with fMRI evidence of selectively observed DG dysfunction), which corresponded to a regionally selective decrease in histone acetylation (itself directly associated with reduced neurogenesis). While Mandel and colleagues interpret their results in terms of normal aging as opposed to AD pathology, our findings tentatively suggest that this mechanism may also be involved in pathological ageing associated with a known CSF biomarker for AD, as well as a known genetic predisposition for the disease.

Drug development for AD is currently moving to earlier manifestations of the disease, that is, the prodromal MCI stage, or even to the preclinical stage. This latter direction has been stimulated by recently published operational research criteria for preclinical AD [24], and therapeutic trials are already underway [25]. Preclinical AD patients are cognitively normal, and thus patients need to be initially identified upon the basis of genetic predisposition or the presence of relevant biomarkers. Further, recent regulatory guidance from the FDA has indicated that in preclinical AD, approval may be given on the basis of cognitive task data alone [26]. The analyses reported in this paper suggest that trials of therapies which target hippocampal neurogenesis in preclinical or prodromal AD may benefit from the targeted selection of participants with reduced CSF Aβ42 and/or those with two ApoE ∈4 alleles. Finally, enhanced DG neurogenesis has been recently shown in animal models to result in improved performance in pattern separation tasks [2]. Thus, in therapeutic preclinical AD trials of compounds, which as (or as part of) their mechanism of action target neurogenesis, pattern separation tasks could provide a proof of mechanism of action, while at the same time serving as part of the assessment of therapeutic effectiveness.

## Conclusions

Mild to moderate AD patients showed a pattern of results on a pattern separation test consistent with disrupted activity in the hippocampal dentate gyrus. fMRI studies have identified that the ability to make difficult object pattern separations in such tests is associated with selectively increased activity in the DG/CA3 region. In this study, the difficult pattern separation measure was selectively impaired in ApoE ∈4 homozygotes, and was also related to CSF levels of Aβ42. These findings are consistent with preclinical findings of a linkage of ApoE ∈4 to compromised DG neurogenesis, and also the established relationship of Aβ to decreased DG neural progenitor cells. These are to our knowledge the first human pattern separation data to suggest a possible genetic link to poor hippocampal neurogenesis in AD, as well as a relationship to Aβ42. Thus, therapies which target hippocampal neurogenesis may be useful in treating the early stages of AD, notably in ApoE ∈4 homocygotes or those showing reduced levels of Aβ42. Further, in such trials, pattern separation tasks could serve both as a proof of mechanism and an efficacy measure.

## Abbreviations

AD: Alzheimer’s disease; ADAS-Cog: Alzheimer’s disease assessment scale cognitive subtest; aMCI: Amnestic Mild Cognitive Impairment; ANCOVA: Analysis of covariance; ApoE: Apolipoprotein E; CDR System: Clinical Dementia Rating System; CI: Confidence interval; CSF: Cerebrospinal Fluid; DG: Dentate gyrus; fMRI: Functional magnetic resonance imaging; MMSE: Mini mental state examination.

## Competing interests

When the study was run, KW was owner of Cognitive Drug Research Ltd, which was contracted to provide the cognitive tests from the company’s proprietary CDR System. The CDR System is now owned by Bracket, which provides services to clinical trials, and KW has been, until recently, an employee and stockholder in the company.

PA was an employee of AstraZeneca, Sweden when the study was run. He is now an employee of Bracket, which provides services to clinical trials.

HB was an employee of AstraZeneca, Sweden when the study was run. He is now employed by BioArctic Neuroscience AB, Sweden.

When the study was conducted, CE was an employee of Cognitive Drug Research Ltd.

KB has served on Advisory Boards for Innogenetics, Roche, Pfizer and Kyowa Kirin Pharma.
